# Organization level research in scientometrics: a plea for an explicit pragmatic approach

**DOI:** 10.1007/s11192-012-0806-6

**Published:** 2012-07-21

**Authors:** Sjoerd Hardeman

**Affiliations:** 1 School of Innovation Sciences, Eindhoven University of Technology, Eindhoven, The Netherlands; 2 Urban and Regional Research Centre Utrecht (URU), Utrecht University, Utrecht, The Netherlands

**Keywords:** Theory of the firm, Unit of analysis, Pragmatism, Epistemology, Classification, Proximity, 90B70, C81, D21, D23, L23

## Abstract

The general aim of this paper is to come to terms with the organization and organization level research in scientometrics. Most of the debate on the issues that revolve organization level research in scientometrics is technical. As such, most contributions presume a clear understanding of what constitutes the organization in the first place. To our opinion however, such “a-priorism” is at least awkward, given that even in specialist fields there is no clear understanding of what constitutes the organization. The main argument of this paper holds that performing organization level research in scientometrics can only proceed by taking a pragmatic stance on the constitution of the organization. As such, we argue that performing organization level research in scientometrics (i) requires both authoritative “objective” and non-authoritative “subjective” background knowledge, (ii) involves non-logic practices that can be more or less theoretically informed, and (iii) depends crucially upon the general aim of the research endeavor in which the organization is taken as a basic unit of analysis. To our opinion a pragmatic stance on organization level research in scientometrics is a viable alternative to both overly positivist and overly relativist approaches as well as that it might render the relation between scientometrics and science policy more productive.

## Introduction

The main aim of this paper is to come to terms with the organization and organization level research in scientometrics. Whatever the exact unit of analysis, by virtue of using bibliometric data, scientometric research is bound to run into data quality issues (Sher et al. 1966; Smith 1981; Moed 1988; Ingwersen and Christensen 1997; Hood and Wilson 2003; Moed 2005). These issues involve among others the completeness, correctness, and interpretability of the data (Galvez and Moya-Anegón 2007). Data quality issues are especially pertinent once we take the organization as the basic unit of analysis (De Bruin and Moed 1990; Bourke and Butler 1996, 1998; Van Raan 2005a, b; Galvez and Moya-Anegón 2006, 2007; Larsen 2008). One notable exception aside (McGrath 1996), most of the debate on this matter is fairly technical. As such, most contributions presume a clear understanding of what constitutes the basic unit of analysis (i.e., the organization) in the first place (see e.g., Van Raan 2005a). To our opinion however, such “a-priorism” is at least awkward, given that even in such specialist fields as economics, economic sociology, and organization and management science there is no clear cut understanding of what constitutes the organization.^1^ This then warrants a discussion on how to conceive of organizations in scientometrics.

The general argument of this paper holds that performing organization level research in scientometrics should proceed by taking an explicit pragmatic stance on the nature and boundaries of the organization. That is, the nature and boundaries of the organization cannot be set purely objective; hence organization level research in scientometrics can only proceed pragmatically. As many “isms” in philosophy, pragmatism has been interpreted differently across the many contributions (Bernstein 2010). Notwithstanding the diversity in interpretations of pragmatism, we center our argument on three main assertions that support our main claim (see also Hjørland and Nissen Pedersen 2005; Hjørland 2008).

The first assertion holds that in order to perform organization level research in scientometrics one is always in need of and indeed always uses some kind of background knowledge on what constitutes an organization. This assertion then is a reformulation of the more general pragmatic claim made already by Peirce (1868) that every cognition is determined by previous cognitions. Perhaps we do not have and in fact even cannot come to a definite understanding of what constitutes an organization; let alone that we are and can be fully explicit about this understanding. Yet this does not mean that we do not have some understanding of the organization on which we might be more explicit. “Comprehending organization level information from bibliometric data: the need for background knowledge” then discusses the need for background knowledge in organization level scientometric research with reference to existing studies available from the literature.

The second assertion holds that performing organization level research involves a balancing between on the one hand classifying the named entities we are confronted with from the bibliometric data as organizations and on the other hand conceptualizing the nature and boundaries of organizations on the basis of our informed intuitions. Performing organization level research then is not just the logic and immediate application of our background knowledge on organizations to the bibliometric data at hand, but also involves an alteration of this background knowledge and hence readjustments in classifying named entities from the data along the way. Comprehending organization level information from bibliometric data then is neither a purely theoretical nor a mere practical job, but reflects a practice which can be more or less theoretically informed. Hence, the more general pragmatic assertion alluded to here is taken from Dewey (1929) and holds that rather than seeking a dichotomy between theory and practice we would rather speak of less informed versus more informed practices in taking the organization as the basic unit of analysis in scientometric research. By drawing upon the discussions on the nature of classificatory work and the nature of the organization, “The boundaries of logic in classification and the logics on the boundary of organizations” discusses why comprehending organization level information from bibliometric data is never a purely logic activity.

The third and final assertion holds that the specific treatment of organizations within organization level research is to be sought in the specificities of the goals, purposes, values, and interests of those pursuing organization level scientometric research. In other words, the way scientometricians make use of bibliometric data for organization level research is thoroughly inflicted with the orientation of the particular studies at stake. This assertion then resonates the idea set out by Putnam (2002) that fact and value are thoroughly entangled. That is, in stressing some aspects and not others in the description or explanation of phenomena lies an inherent attachment to particular normative positions. In “How goals and interests feed into organization level research in scientometrics” then we continue to discuss our personal experience in using bibliometric data for organization level scientometric research. In so doing we try to make clear how the goals, purposes, and interests of our own study fed into our classification of organizations. Final section concludes with some general remarks on some of the implications of this paper.

## Comprehending organization level information from bibliometric data: the need for background knowledge

It is often stressed that using the organization as a basic unit of analysis in scientometrics requires a lot of cumbersome work in cleaning the bibliometric data (Moed 1988; De Bruin and Moed 1990; Moed et al. 1995; Bourke and Butler 1996, 1998; Van Raan 2005a, b). Part of this cumbersome work not only applies to the organization as a basic unit of analysis, but applies to other units of analysis in scientometrics as well. In general then, whatever the particular unit of analysis, bibliometric data suffer from many inconsistencies across records (Sher et al. 1966; Smith 1981; Moed 1988, 2005; Ingwersen and Christensen 1997; Hood and Wilson 2003).

One of the main problems associated with extracting organization level information from bibliometric data is called the unification problem (see e.g., Moed 2005, pp. 183–187). That is, the problem that information pertaining to a single organization is scattered across multiple records in different forms. The problem of unification is basically twofold (Galvez and Moya-Anegón 2007). First, there is a lack of consistency in naming organizations across entities. Thus, the same organization is named differently across entities; i.e., bibliometric data contain organizational synonyms. Alternatively, not only do bibliometric data contain synonyms, they also contain homonyms; across records the same named entity can refer to different organizations. Second, there is a lack of consistency in the amount of and the order in which named entities occur across records. That is, while some records contain a host of named entities containing information on all kinds of organizational aspects (e.g., “University of California at San Diego; School of Medicine; Department of Epidemiology; Division of Cardiovascular diseases; San Diego CA; United States”), other records make mention of a restricted number of named entities only (e.g., “University of California; San Diego”). What is more, the order in which these named entities occur across the data need not be standardized. That is, while some records might mention named entities belonging to the main organization first (e.g., “University of California; …; etc.”), other records might mention named entities belonging to the sub organization first (e.g., “School of Medicine; …; etc.”).

Whatever the specific causes of this twofold unification problem, what holds is that one cannot proceed in solving it without the use of background knowledge on what constitutes an organization. The need for background knowledge is readily acknowledged in the literature discussing the problem of unification. Moed (2005 p. 185) even explicitly states that “*[b]ackground knowledge about the institutions is essential*”. However, most of the—implicit or explicit—references made to the need for background knowledge seem to refer to a particular kind of background knowledge. That is, most accounts on the need for background knowledge seem to refer to a need for a dictionary or other authoritative communications making sure that the named entities on organizations being scattered in different forms across multiple bibliometric records are justly unified. As such, Moed (2005 pp. 185–186) goes as far as to argue that “*an appropriate identification scheme of an organisation’s publication output must involve detailed background knowledge provided, or at least thoroughly checked by, the organisations themselves. Verification by representatives of the organisations is indispensable for obtaining outcomes that are sufficiently accurate and hence can be properly used in policy analysis and the public domain.*” The point is however that rather than solving the problem of justly unifying named entities, most contributions seem to merely circumvent the problem with reference to authority.

In fact, in referring to a need for authority as a kind of background knowledge, at least three additional problems are being introduced. One pertains to the problem of what constitutes an authority in the first place. In other words, which dictionary or communication counts as an authority and which not? Related, another problem pertains to ascertaining the basis of the authority diverted to. Now, without having the pretention to solve these problems here, what holds is that they all point at the need for additional background knowledge that precedes our use of authoritative background knowledge such as dictionaries and communications with knowledgeable people. Obviously, these three problems cannot be completely solved by introducing more authoritative background knowledge, for these authorities would in turn require additional background knowledge to be interpreted and hence would eventually leave us being stuck in an infinite regress (cf. Collins 1985).

To make our point clear, consider an example from our own research in which bibliometric data records mention “Steno Diabetes Center; Copenhagen; Denmark”. From its name “Steno Diabetes Center” alone it is not clear at all what kind of organization this is. Hence, a priori we cannot know whether this named entity should be treated as a single organization or whether it belongs to another main organization. In order to solve this ambiguity we turned to the website of this entity. That is, we made use of what we consider to be an authoritative source on the nature and boundaries of this named entity. From their website we read among others the following (Steno Diabetes Center 2011): “*Steno Diabetes Center is a world leading institution within diabetes care and prevention. Steno is owned by Novo Nordisk A/S and is a not for profit organisation working in partnership with the Danish healthcare system. … Steno Diabetes Center is associated with the University of Copenhagen through the university’s hospitals management forum … Our vision is to become leaders in diabetes care and translational research with focus on early disease and prevention.*” In itself however, these excerpts do not provide in a conclusive idea on the objective status of “Steno Diabetes Center” as an organization. In order to provide in such a conclusive idea then we need to make additional judgments grounded in some kind of background knowledge that goes beyond these statements alone. The point we would like to stress then is not that the rules that have been used throughout the literature so far are necessarily wrong, but rather, and more modestly, that we need such rules in the first place if we are to perform organization level research using bibliometric.

The extent to which inconsistencies in bibliometric data are problematic for performing scientometric research depends on the nature of the analysis. In general one can distinguish between top-down scientometric analysis and bottom-up scientometric analysis. In top-down scientometric analysis the researcher starts from the science system as a whole (i.e., including all publications at first) and subsequently has to sort out which publication belongs to which organization. In bottom-up scientometric analysis the researcher starts from information on individual organizations and only then searches for organizational data. Obviously, it is more straightforward to collect most if not all the publications of a single organization (as in bottom up scientometrics) than assigning a large set of publications to many different organizations (as in top-down scientometrics). However, the more a researcher positions a single organization in the broader science system, the more one moves away from performing bottom-up scientometrics towards performing top-down scientometrics. As such, scientometric researchers do not only need to have additional background knowledge, they also need to decide where and how far they will go in collecting such fine grained organization level data.

What holds then is that the nature and boundaries of the organization do not follow immediately from the bibliometric data itself. Rather, the nature and boundaries of the organization have to be imposed on the data by the researcher using it. Indeed, we cannot filter out organization level information from bibliometric data alone but need additional background knowledge. However, the point we would like to raise is that the particular background knowledge that we need pertains but cannot be restricted to the use of authoritative sources because these sources would require additional background knowledge to be understood and further applied in turn. Non-authoritative background knowledge then remains a prerequisite for any idea on organizations one starts off with and continuous to be necessary in further substantiating and adapting one’s idea on the nature and boundaries of organizations as they are represented within bibliometric data.

## The boundaries of logic in classification and the logics on the boundary of organizations

### Classification and the boundaries of logic

The previous section of this paper discussed why background knowledge is always necessary if we are to comprehend organization level information from bibliometric data. Hence, we always need and indeed always use background knowledge on what constitutes an organization in order to comprehend organization level information from bibliometric data. As a first approximation then let us define the organization as follows: an organization is a group of people and their resources together performing tasks to achieve a common goal (e.g., Parsons 1956a). The task of comprehending organization level information from bibliometric data can then be characterized as on the one hand involving classificatory work and on the other hand involving conceptual work. In what follows we will discuss both in turn.

The idea of classification involves at least three aspects (Spärck Jones 2005). First, by implication, any classification is supposed to divide the universe of entities into a smaller number of objects. If we would keep the range and number of entities as they appear we cannot speak of a classification in the first place. With respect to our concern here we are concerned with a reduction of all named entities into sensible organizations. The underlying rationale of every classification then is to provide in a simplification for the complete range of different entities. Second, any classification is based on the premise that any two entities that appear within the same class can be said to be similar in one way or another. That is, entities that belong to the same class (i.e., organization) share characteristics that make them distinct from other classes (i.e., organizations). Third, any classification is meant to attribute meaning to the classes thus derived. That is to say, by virtue of assigning an object to one class and not to another this object gets a particular interpretation and not another. Without such meaning classifications can be said to reflect mere groupings of objects and as such can readily be conflated with statistical techniques such as clustering. Such statistical techniques however in itself never provide in an interpretation of these groupings, something that classifications do strive for. More formally then, we can describe classifications as meaningful groupings of objects that resemble each other (see also Hjørland and Nissen Pedersen 2005).

Ideally then, we would like to come up with a classification system in which all entities can be consistently and meaningfully assigned to mutually exclusive classes (Bowker and Star 1999). Developing such an ideal type classification is however constrained by issues of logic, issues associated with meaning, and the interaction between these issues of logic and meaning (Hjørland and Nissen Pedersen 2005). First, logical issues revolve around the extent to which entities can be systematically, exhaustively, and non-discriminatory assigned to classes on the basis of their properties. Depending on the number of properties characterizing each entity and the number of organizations we are to deduce from these objects it can be readily shown that a logic classification need not be possible. Consider for example 3 entities (I, II, III) with each two properties (A and B) that can be of two types (A1 vs. A2 and B1 vs. B2).^2^ If element I is characterized by properties A1 and B1, element II is characterized by properties A1 and B2, and element III is characterized by properties A2 and B1 we cannot logically deduce 2 organizations from these three elements. Either we favor property A over property B and we consider elements I and II as one organization leaving element III as a second individual organization or we favor property B over property A and we consider elements I and III together as one organization leaving element II as a second individual organization. Again Spärck Jones (2005) argues that the more a classification can be characterized as polythetic, overlapping, and unordered, the less feasible a logic classification becomes.

Second, issues of meaning revolve around the interpretation of different classes in terms of their representative function. Consider the possibility that the organization itself might be thought of in different terms by different people. Consider again 3 different entities (I, II, III) but now each with four properties (A, B, C, D). Obviously, if some only take properties A and B as constitutive characteristics of organizations therewith disregarding properties C and D while others conceive of properties C and D as constitutive characteristics of organizations therewith disregarding properties A and B we end up with different organizations if properties A, B, C and D are distributed differently (i.e., do not come in pairs) across entities. If for example entity I is characterized by properties A1, B1, C2, and D2; entity II is characterized by properties A1, B1, C1, D1; and entity III is characterized by properties A2, B2, C1, and D1; it follows that these entities will form different organizations depending on which properties are deemed important in constituting an organization. Here, Mai (2004) rightly argues that any characterization of units (i.e., organizations) in terms of properties depends crucially upon what counts as a constitutive property and hence how organizations ought to be thought of in the first place.

Third, the interaction between issues of logic and meaning revolve around situations in which these two issues might be in conflict. On the one hand, based on a given set of properties a logic classification might be deductible whose classes can be said to have little meaning. For example if properties A1 and A2 are distributed evenly across a large amount of entities, classifying on the basis of this property only might render large chunks of objects we would hardly call organizations. In other words, a particular property might be a necessary but not a sufficient condition (i.e., a defining characteristic) to call a group of entities an organization. Such properties then are not considered distinctive enough to base a meaningful classification scheme on.

On the other hand, a meaningful identification of classes need not be logically deducible due the possible transgressive nature of the properties involved. That is, while the boundaries of the prime unit of interest may be vaguely set, so can the properties that are said to make up this main unit. With respect to comprehending organization level information from bibliometric data, and anticipating our discussion of the boundaries of the organization in the next section, we argue that many of the characteristics defining the organization are fluid rather than fixed. That is, many properties characterizing the organization gradually flow into its surroundings.

To conclude on classificatory issues, we stress that although in principle we might be able to come up with a logic classification of organizations from information entities available in bibliometric data, this classification might not make sense in terms of our general idea on what constitutes an organization. Hence, in classifying objects from a set of elements often we have to find a middle way between interpretative richness (i.e., including all possible properties in their variegated appearances) and logical robustness (i.e., relating elements systematically to form coherent objects). This then brings us at discussing our basic idea on what constitute the nature and boundaries of the organization beyond our first approximation given at the beginning of this section.

### The logics of the boundary of the organization

Let us first qualify the classification issue at hand a bit more formally. Strictly when we speak of classifying named entities that refer to organization level information from bibliometric data we do not necessarily talk about the classification of organizations around us. That is to say that we only speak about the organizations as they are represented by particular information entities in bibliometric data. In principle we are interested in classifying these named entities into meaningful groups that we call organizations instead of being interested in classifying organizations as such. To the extent that we concordate named entities from bibliometric data that refer to organization level information with a given set of organizations that we see around us, one might even argue then that we are concerned with matching information rather than classifying organizations. Yet, to the extent that we don’t know what it is that we call organizations as we see them around us we are not just matching organizations as they exist but also classifying them at the same time. This then immediately brings into focus classifying organization level information from bibliometric data as problematic given its non-straightforward interpretation as a basic unit of analysis in the first place.

Already from an intuitive understanding on the nature and boundaries of the organization we can come up with numerous aspects that can be considered as belonging to the organization. That is, defining the organization as constituting a group of people and their resources together performing tasks to achieve a common goal, immediately brings to the fore a number of images on the organization (Morgan 1986). As such, we can link the nature and boundaries of the organization to a particular name (e.g., Eli Lilly and Company), a particular good (e.g., the drug Prozac) or abbreviation (e.g., its ticker symbol LLY), but also to an exemplary building and its location (e.g., the Lilly Corporate Center in Indianapolis, Indiana), a particular subsidiary (e.g., e.g., Elanco Animal Health), an even finer grained organization level (e.g., Lilly Research Laboratories) or just an individual (e.g., its CEO John C. Lechleiter).

Although all important in their own right, these images together do not immediately provide in a comprehensive picture of the organization as a whole. Yet they do provide with some insights on the constitutive characteristics of the organization. One such characteristic pertains to the organization as defined by legal ownership structures. The ILL ticker symbol, Elanco Animal Health as a subsidiary of Eli Lilly and Company, and John C. Lechleiter being its CEO all very much resonate an idea of the organization as a formal legal entity and relates to descriptions of organizations as hierarchical, bureaucracies, and involving employment relationships controlled by managers. In a very strict sense, organizations ought to be distinguishable from one another through clearly identifiable distinct hierarchical control systems (i.e., ownership and employment relationships). This idea is particularly salient within transaction costs economics approaches to organizations (see e.g., Williamson 1981). The argument from the classic contribution of Coase (1937) holds that the existence, size and boundaries of the organization (firms in his account) are determined by its relative efficiency to coordinate exchanges as compared to the market. As long as hierarchical/managerial control is more efficient (i.e., less costly) in coordinating exchanges than prices, the formal organization will be the dominant mode of coordinating economic activities. In the context of drawing organization level information from bibliometric data here, this would imply taking into account the legal ownership status of every organizational constellation vis-à-vis other organizational constellations.

Another constitutive characteristic of the organization revolves around the kind of activities performed by the organization. Producing pharmaceutical drugs is thus taken as a constitutive characteristic of Eli Lilly and Company. The good or range of goods that are produced often contrasts one organization from another organization. As such, the goods produced by a commercial firm are often taken as different from the goods produced by a university. Not only then, are the goods taken as different but also the means by which these goods come about are very much constitutive of the organizations producing them. For that matter, commercial firms are said to operate by a different set of norms and values than universities. The idea of organizations as delineated by the kind of and way they produce goods, very much resembles the idea of Parsons (1956a) on organizations as functionally and institutionally differentiated subsystems of society. As such, Parsons (1956b) identifies four such subsystems which can in principle be further differentiated: (i) organizations with an economic goal orientation, (ii) organizations with a political goal orientation, (iii) integrative organizations, and (iv) pattern-maintenance organizations. Apart from taking into account the legal ownership status of organizational constellations then, a view on organizations as constituting functionally and institutionally differentiated subsystems of society, implies that in cleaning up organization level bibliometric data we should also take into account what and how these organizational constellations produce goods.

A final characteristic discussed here pertains to a connotation of the organization as a place or space. This place can be a very concrete building such as the Lilly Corporate Center or a more abstract space as for example Lilly Research Laboratories. This place can be fairly concentrated such as in the city of Indianapolis (Indiana) or very distributed as is inherent to the idea of Eli Lilly and Company as a multinational. What holds then is that the idea of organization has a clear geographical connotation (see also Dicken and Malmberg 2001). As such, a geographical connotation to organizations refers to co-presence as a constituting characteristic of as organizations defined earlier as groups of people and resources jointly performing tasks to achieve a common goal. More in general then, geography provides in a means to set the boundaries of the organization; i.e., a means to bundle labor, resources, and markets (to the extent that these are locally constituted). What is more, many named entities in bibliometric data contain information on the locality of organizations and hence provide in the means to actually nail down organizations on the global map (Leydesdorff and Persson 2010).

So far, our understanding of the nature of the organization is very much directed at the organization as a multidimensional study object. Apart from the already discussed dimensions legal ownership, type of activities involved, and geographical scope, other dimensions can be easily added such as the knowledge base.^3^ However, whatever the exact dimensions involved, this multidimensional nature in itself does not tell much about the scope of these dimensions, that is, their boundaries. For some dimensions the exact boundaries of the organization seem to be easily set in theoretical terms. With respect to legal ownership for example it can be readily argued that the boundaries of the organization can be drawn at the point where its power to execute formal control stops. Likewise, the boundaries of the organization can be drawn at the point where different goals are pursued.

These theoretical ideals are however hardly systematically tenable once we need to comprehend organization level information from bibliometric data empirically. First, the idea that organizations can be distinguished on the basis of legal ownership and employment relationships make organizations that are intuitively taken as distinct (e.g., Eli Lilly and Company and Pfizer, Inc.) potentially to be considered as one organization by virtue of their boards being interlocked (see e.g., Mizruchi and Schwartz 1992). What is more, organizations that are intuitively taken as distinct might also be linked via formal partnerships (e.g., via alliances) and cross-ownership (see e.g., Shleifer and Vishny 1997). Second, the idea that organizations can be distinguished on the basis of their activities only shifts the issue to identifying mutually exclusive activity categories, that is, to answering such questions as does “university X” perform the same activities as “Research Institute X”? In addition, ascertaining the geographical scope of an organization need not be straightforward as well. That is, many organizations, as we intuitively understand them, are scattered across a larger industrial site, a city, regions, and even countries. Hence the idea of the organizations as belonging to a particular point on a geographical map does not necessarily hold.

In all then, and despite an understanding of the nature of the organization as revolving multiple dimensions, we are not able to fix the boundaries of the organization unambiguously. Rather, what we are left with is an understanding of the organization as in itself reflecting a multi-dimensional network among a dense web of relationships (Badaracco 1991). The task of comprehending organization level information from bibliometric data as on the one hand involving classificatory work and on the other hand involving conceptual work is hence problematic for at least three reasons. First, the nature of the organization can be characterized along multiple dimensions. Although not problematic in itself, this makes ideal type classificatory work in organization level scientometric research highly unlikely. Second, and more problematic, is that the scope of the dimensions defining the nature of the organization cannot be objectively fixed. That is, at some point we might speak of some entities as belonging more or less to any particular organization; however the exact point at which the one organization ends and the other begins cannot be set completely unambiguously. As such, the nature of the organization can be characterized as thoroughly transgressive leaving an unambiguous assessment of organizations in scientometrics virtually impossible. All this does not imply that we cannot perform organization level research in scientometrics altogether. Rather, and without falling into a trap of mere subjectivism, it is to suggest that we should abandon the sometimes salient idea in scientometrics that organizational level research herein can be completely objective.

## How goals and interests feed into organization level research in scientometrics

The previous section of this paper discussed the practice of performing organization level research in scientometrics as involving a balancing act between on the one hand classifying the named entities we are confronted with from the bibliometric data as organizations and on the other hand conceptualizing the nature and boundaries of organizations on the basis of our informed intuitions. Both classification and conceptualization then run into the limits of logic; along the way of comprehending organization level information from bibliometric data we have to make ad hoc decisions at some point. This section pursues this argument further and addresses how views upon the organization reflect the goals and interests of organization level research in scientometrics. In order to make this argument clear we will draw primarily upon our own organization level research and compare this with other studies from the literature.

In the study for which we use organization level information from bibliometric data we are interested in a characterization of territorial science systems (see Hardeman et al. 2012). Following the notion of innovation systems (Carlsson et al. 2002; Lundvall 2007), we first defined science systems as a set of interacting organizations. A characterization of territorial science systems then involves typifying these organizations and their interactions. Different contributions to the literature on science and innovation systems stress different aspects in the characterization of organizations and their interactions (see among others Lundvall 1988; Gibbons et al. 1994; Etzkowitz and Leydesdorff 2000; Bonaccorsi 2008). Of all these contributions we believe that the notion of Mode 2 knowledge production (Gibbons et al. 1994; Nowotny et al. 2001) provides in a fairly inclusive account in that it addresses an extensive number of dimensions at once (see also Hessels and Van Lente 2008). Hence we sought to characterize territorial science systems along five dimensions they pay attention to; i.e., the geographical, cognitive, social, institutional, and organizational dimension to knowledge production (see also Frenken et al. 2009). As such we had to characterize every organization in our data as a point in multidimensional space.^4^

In order to comprehend organization level information from the bibliometric data and assign to a point in multidimensional space we followed a five-step procedure (see also the “Appendix”). First we collected the data pertaining to our science system of interest, that is, we collected all bibliometric records that represent publications concerned with tackling the problem of type 2 diabetes. Second, from the data thus retrieved we extracted all named entities we deem important as possibly pertaining to information on the organizations of interest. Third, in order to make sure that unique organization IDs are consistently attributed across publication records and can thus be used as a starting point for our classification work, we manually checked a random set of organization id’s for internal consistency. Fourth, we formulated a set of three rules that can be used to comprehend organization level information from bibliometric data. Fifth and finally, we applied these rules accordingly using two extra sources (i.e., the organizations’ websites and an online tool for attributing geographical coordinates to the organizations).

The way goals and interests feed into the classification of organizations is best illustrated through a more in-depth discussion of the rules we applied in comparison to those applied by others in their classification work. Note however that the requirement of background knowledge discussed in “Comprehending organization level information from bibliometric data: the need for background knowledge” of this paper already comes in when for example manually checking organization IDs on their internal consistency in step two and three. This consistency can only be checked against the background of some kind of baseline and thus requires considerable interpretation which in turn can only be performed with some kind of background knowledge. Likewise, the application of the rules set in step five requires considerable interpretation and as such can again only be performed with the use of background knowledge. It is however in the actual formulation of classification rules and its underlying rationale that our goals and interests come most to the fore.

In order to comprehend organization level information for purposes of assessing territorial science systems we applied a set of three rules (see “Conceptualizing organizations: formulating rules to unify strings” of the Appendix). The first rule sets the hierarchical boundaries of the organization; the second rule sets the institutional boundaries of the organization; and the third rule sets the geographical boundaries of the organization. Together then we take the organization as a bundle of boundaries revolving on hierarchy, institutional domain, and geography (see also Carlile 2004 on the organization as a bundle of boundaries).

The geographical rule is perhaps a most obvious instance in which our study concern feeds into our classification of organizations. That is, if we are to assess interactions between any two organizations in terms of their geographical proximity (or distance), we have to locate every organization (as we define them) on the world map. This is not to say that assigning organizations to a particular location is a straightforward job to do. For one thing, a single organization can be substantively geographically distributed. While some organizations are located in a single building, other organizations are spread across a city, country or even the entire world. Second, the way the location of an organization is inscribed on publications and subsequently represented within publication records might vary considerably across publication records for the same organization. In order to still be able to assign every organization to a single location part of our classification depends on restricting the geographical scope of any organization by a maximum of 50 km separation between any two elements pertaining to the same organization id.^5^ Whereas in assessing territorial science systems every organization has to be attributed to a particular geographical location, in ranking organizations no such reference to the location of the organizations involved. That is, in ranking for example companies’ productivity in terms of publication output it does not matter whether a particular company has branches on multiple locations across the world or is located at one particular site only. Hence while in some studies the geographical dimension need not be taken into account per se, for purposes of assessing territorial science systems the geographical dimension of the nature and boundaries of the organization have to be taken into account by implication.

With respect to our institutional identity of organizations we deliberately decide not to assign publications to organizations that are not mentioned on the publication record itself. Compare this with Van Raan (2005a) who argues from his concern with ranking universities that for some organizations (like CNRS in France) the publications should be assigned to a university. Likewise, in ranking universities the publications of hospitals neighboring the universities should also be assigned to the latter (especially university hospitals). Our concern however specifically resides in an assessment of the heterogeneous nature of interactions between organizations. As such it would be questionable to assign publications of for example political agencies or hospitals to universities especially given that claims on “Mode 2 knowledge production” (Gibbons et al. 1994; Nowotny et al. 2001) and a “post-modern research system” (Rip and Van der Meulen 1996) emphasize the role played by organizations other than universities. If we are to include these non-traditional scientific knowledge producing organizations into our analysis we cannot assign publication records of them to universities. All this is not to suggest that the method advocated by Van Raan (2005a) and others is misguided per se. On the contrary, given the “*fiercely debated*”, “*sometimes controversial*”, and “*politically highly sensitive*” nature of university rankings (Moed 2005, p. 185), it is perhaps advisable to go for a strategy that reduces type 2 errors to a minimum (i.e., not ascribing some publications to an organization while in fact they should have been). What holds, however, is that there is nothing natural or logical in attributing for example the output of university hospitals to a particular university.

We do not claim that our approach in classifying organizations is the best, let alone that our approach is most suitable for different kind of uses of organization level scientometric research. On the contrary, what we like to stress is that our classification of organizations has to be seen in light of the general orientation of our study. Given that both science systems and organizations can be described in similar terms along multiple dimensions we have to set boundaries on where the organization stops and the outer system begins. These boundaries then can be set reasonably, but not pure logically. To generalize on this point, we believe that every classification of organizations used in scientometric studies has to be seen in light of the goals, purposes, and interests of these studies. That is, throughout classificatory work in organization level scientometric research, scholars have to make choices. This does not mean that these choices are made arbitrary; most scholars have good reasons to go for one particular way of comprehending organization level information and not another. Yet, the choices that we make cannot be qualified as either “best” or “thoroughly wrong”; rather the choices that we make are biased, fallible (not wrong per se!), and hence always open to debate.

## Concluding remarks

The main aim of this paper was to come to terms with the organization and organization level research in scientometrics. We deem this a pertinent issue, given that organization level research in scientometrics is abound although the whole notion of what constitutes an organization is rather vaguely set. It seems that in identifying unique organizations most scientometric studies thus far apply a set of mostly implicit rules only that appear to be objectively set. As argued throughout this paper however, rather than being objectively set, the boundaries of the organization in scientometrics can only be set pragmatically.

Our discussion of the pragmatic nature of organization level research in scientometrics might give the impression that we think of pragmatism as a theory instead of a philosophy, that is, as a way in which scientometrics actually proceeds rather than how scientometrics ought to proceed. Indeed, to our opinion, and as we have tried to show throughout this paper, organization level research in scientometrics can only proceed pragmatically. This claim of course leaves open the issue whether this situation is applaudable or not.

Let us then, by way of conclusion, briefly reflect on the normative implications of our main claim. For at least two reasons an explicit pragmatic approach to organization level research in scientometrics need not be lamentable. First, pragmatism opens the door to theoretical and methodological pluralism in scientometrics. An explicit recognition of the non-foundationalist and fallible nature of research might render non-positivist approaches more viable. This is certainly not to propose a relativist approach to scientometrics (see also Mäki 1997; Collins 2009). Rather, it is an appeal on taking the provisional nature of all knowledge claims seriously. This means amongst others that scientometric studies have to open up on its conceptual, theoretical, and methodological proceedings (see also Opthof and Leydesdorff 2010). To our opinion then, explicitly recognizing the non-foundationalist and fallible nature of (social) scientific knowledge claims increases the likelihood that scientometrics comes up with a range of viable solutions to the issues at stake.

Second, an explicit pragmatic stance on organization level research in scientometrics might also help scientometrics to come to terms with those using its outcomes. As a specialist field of research, scientometrics easily runs the risk of being used uncritically by science policy makers and the lay public (Van Raan 2005a; Weingart 2005). Rather than seeking the solution to this problem only at the side of those using scientometric research, we believe there is much to gain once scientometrics itself becomes more open about its proceedings and practices (see also Shapin 1992). Given that organization level research in scientometrics can never be purely objective, scientometrics might consider being more explicit on its fallibility possibly rendering awareness at the side of science policy makers that scientometrics can indeed not be used uncritically. In all then, we not only believe that much of organization level research in scientometrics actually proceeds pragmatically; we also believe that an explicit pragmatic stance in scientometrics is a viable alternative to both overly positivist and overly relativist approaches as well as that it might render the relation between scientometrics and science policy more constructive.

## Appendix

### Extracting bibliometric records representing publications on type 2 diabetes

Extracting bibliometric information pertaining to a particular research field or discipline is in itself far from straightforward. Just as the organization is a highly transgressive entity, so are disciplines, research fields, and even—as in our case—particular research topics. The particular issue at hand involves coming up with a set of search terms that are both general enough to extract all records reflecting upon research on type 2 diabetes and still specific enough in order not to extract records that are not concerned with type 2 diabetes at all. The issue is especially complicated given that the arsenal of terms that is used to describe diseases (like type 2 diabetes) changes over time and across contexts (for a discussion on this matter see Bowker and Star 1999). As such, the whole term type 2 diabetes as a particular form of diabetes for example did not even exist 70 years ago (Tattersall 2009). However, in restricting ourselves to a specific and fairly narrow time frame (1996–2008), we believe we are still able to come up with a comprehensive set of terms that capture type 2 diabetes during that period.

We used Elsevier’s Scopus database to extract bibliometric records concerned with type 2 diabetes. In order to identify and extract all bibliometric records representing documents that are concerned with research on type 2 diabetes we constructed a search query based on a list of tags that capture the different names used to address this health problem (see Table 1). The list that we used is adapted from discussions that we had with experts from this field of research and is complemented by terms denoting type 2 diabetes as they are provided in medical classification systems of the International Classification of Diseases (World Health Organization 2011), the Medical Subject Headings (MeSH) (U.S. National Library of Medicine 2011), and EMTREE (Elsevier Pharma Development Group 2009). Using the search query thus defined, we extracted 72,725 uniquely coded bibliometric records that represent scientific publications concerned with type 2 diabetes for the period 1996–2008.

**Table 1:** Search query to extract publication records on type 2 diabetes

1. The source of our bibliometric data is the offline version of Elsevier’s Scopus which we acquired in June 2009
2. In order to retrieve records representing evidence from research on type 2 diabetes we searched for records mentioning in one way or another the following terms in their abstract, title or (indexed or author) keywords: “non insulin dependent diabetes”, “adult onset diabetes”, “mason type diabetes”, “maturity onset diabetes”, “insulin independent diabetes”, “non ketotic diabetes”, “stable diabetes”, “type 2 diabetes”, “type ii diabetes”, “ketosis resistant diabetes”, “slow onset diabetes”, “mody”, “lipoatrophic diabetes”, “insulin independent diabetes”, “dm2”, and “niddm”
3. Note that some terms denoting type 2 diabetes research are rather general, that is, some terms are suspect of having meanings not referring to type 2 diabetes in specific (e.g., “dm2”). Hence, we first performed a more general search for diabetes research using “diabetes” and “diabetic” as search terms only
4. More formally then we used the following search query:
{{{diabetes OR diabetic} AND {{adult onset} OR {adultonset} OR {adult-onset} OR {auto somal dominant} OR {autosomal dominant} OR {auto-somal dominant} OR {autosomaldominant} OR {autosomal-dominant} OR {auto-somal-dominant} OR {insulin independent} OR {insulinindependent} OR {insulin-independent} OR {ketosis resistant} OR {ketosisresistant} OR {ketosis-resistant} OR {late onset} OR {lateonset} OR {late-onset} OR {mason type} OR {masontype} OR {mason-type} OR {maturity onset} OR {maturityonset} OR {maturity-onset} OR {non insulin dependent} OR {non insulindependent} OR {non insulin-dependent} OR {non ketotic} OR {noninsulin dependent} OR {non-insulin dependent} OR {noninsulindependent} OR {non-insulindependent} OR {noninsulin-dependent} OR {non-insulin-dependent} OR {nonketotic} OR {non-ketotic} OR {slow onset} OR {slowonset} OR {slow-onset} OR {type 02} OR {type 2} OR {type ii} OR {type-02} OR {type-2} OR {type-ii} OR {aodm} OR {dm 2} OR {dm2} OR {dm-2} OR {mod} OR {mody} OR {ncdmm} OR {niddm} OR {niddy} OR {aodm,} OR {dm 2,} OR {dm2,} OR {dm-2,} OR {mod,} OR {mody,} OR {ncdmm,} OR {niddm,} OR {niddy,} OR {aodm:} OR {dm 2:} OR {dm2:} OR {dm-2:} OR {mod:} OR {mody:} OR {ncdmm:} OR {niddm:} OR {niddy:} OR {aodm;} OR {dm 2;} OR {dm2;} OR {dm-2;} OR {mod;} OR {mody;} OR {ncdmm;} OR {niddm;} OR {niddy;}} OR {{stable diabetes} OR {stable diabetic} OR {diabetes, stable} OR {diabetic, stable} OR {stable-diebetes} OR {stable-diabetic}} OR {{diabetes in young} OR {diabetes in youth} OR {diabetes mellitus in young} OR {diabetes mellitus in youth} OR {diabetes mellitus of the young} OR {diabetes mellitus-in-young} OR {diabetes mellitus-in-youth} OR {diabetes mellitus-of-the-young} OR {diabetes of the young} OR {diabetes-in-young} OR {diabetes-in-youth} OR {diabetes-mellitus in young} OR {diabetes-mellitus in youth} OR {diabetes-mellitus of the young} OR {diabetes-mellitus-in-young} OR {diabetes-mellitus-in-youth} OR {diabetes-mellitus-of-the-young} OR {diabetes-of-the-young} OR {diabetic in young} OR {diabetic in youth} OR {diabetic of the young} OR {diabetic-in-young} OR {diabetic-in-youth} OR {diabetic-of-the-young} OR {diabetics in young} OR {diabetics in youth} OR {diabetics of the young} OR {diabetics-in-young} OR {diabetics-in-youth} OR {diabetics-of-the-young}} AND {{maturity onset} OR {maturityonset} OR {maturity-onset} OR {non insulin dependent} OR {non insulindependent} OR {non insulin-dependent} OR {noninsulin dependent} OR {non-insulin dependent} OR {noninsulindependent} OR {non-insulindependent} OR {noninsulin-dependent} OR {non-insulin-dependent}}}

## Extracting information on organizations from bibliometric records

Every record represents one or more strings of information. These strings are divided by 8 named entities pertaining to organization level information (see Table 2). The different named entities contain information on (i) the main name, (ii) a main organization ID, (iii) a sub-name, (iv) a sub-ID, (v) a country location, (vi) a city and/or region location, (vii) a more fine grained description of the location of an organization (e.g., a street, zip-code or post box; we call this the address), and (viii) additional organization level information not attributed to any of the other six named entities (we call this rest information). If we record wise split these strings of named entities we identify 186,719 such publication-record/organization-information combinations.

**Table 2:** Strings with named entities (186, 719 strings in total; 8 named entities per string)

Main name	Main ID	Sub name	Sub ID	Country	City	Address	Rest

## Classifying organizations (I): scopus’ main organization IDs as a starting point

In comprehending organization level information from our bibliometric dataset we choose to start from the main IDs. Given that within 96 % of all strings the named entity on the main ID contains information at all and that the total number of different main IDs is relatively small (i.e., 22,647 main organization IDs); our unification problem would be considerably reduced if these main IDs are consistently attributed across all strings. In order to make sure that Scopus assigned the main IDs consistently, we randomly checked 105 such id’s which occur across 18,390 strings (9.8 % of all strings). This manual checking involved making sure that the different main names attributed to each unique ID are enough similar to conclude that they indeed represent the same organization. We performed this checking manually and conclude that, whereas only 1.8 % of all ID-name combinations represent deviating main names, in general Scopus’ main organization IDs are consistent across strings. This then lends support to taking Scopus’ main organization IDs as our starting point in comprehending organization level information from the bibliometric data at hand.

Note however that the assertion that the main organization IDs within our dataset are internally consistent does not imply that they immediately leave us with a coherent set of entities which can reasonably be said to represent information on unique organizations. First, multiple main organization IDs might refer to the same organization entity. Most straightforward then, there might be two IDs both pertaining to the main organization name “Harvard University”. Likewise, there might be two IDs that pertain to the same organizations; e.g., one referring to “Leiden University” and the other referring to “University Leiden”. Second, a single main organization ID might also (consistently) refer to multiple organizations (or organizational entities). For example, the name “Harvard Medical School Boston Brigham and Women’s Hospital” pertains consistently to a single main organization ID but can be reasonably considered to belong to two different organizations; i.e., Harvard Medical School and Boston Brigham and Women’s Hospital. Hence, we still need to comprehend (unify and split) the main organization IDs to render unique organization level information that makes sense.

In addition there are two other issues to take into consideration. One is that locational information is attributed inconsistently across strings pertaining to the same main organization ID. As such, the named entity reflecting the city name of an organization might refer to different cities for the same main organization ID. For example, the main organization ID referring to the University of California might sometimes refer to Los Angeles as its city location while in other cases it refers to San Diego. Likewise, the main organization ID of a multinational organization might report on locations across multiple countries. What is more, the detailed description of locational information differs across cases belonging to the same main organization ID. Thus, while it is possible to locate some cases at the address level, for other cases of the same main organization ID we only have locational information at the city level. Second, the hierarchical level to which a main organization ID pertains differs across main organization IDs. As such, “Harvard University” belongs to a particular main organization ID while “Harvard University Medical School” belongs to another main organization ID. Hence, main organization IDs can in principle refer to different levels of the same organization.

Table 3 summarizes the main problems of unification: (i) a main name (X) can be scattered across multiple IDs (1 and 2); (ii) a main ID (3) can refer to multiple organizations (X and Y); (iii) a main ID (4) can be scattered across multiple cities (A and B), multiple countries (i and ii), and multiple addresses (a and b). Note that we choose not to take the named entities sub name, sub ID, and rest information to comprehend organization level information. Of these named entities, the sub ID had a coverage of only 72 %. Yet for those instances for which we do not have a main ID at our disposal (i.e., 4 %) we manually attribute a main ID judged on the basis of information contained by other named entities including these three.

**Table 3:** Problems of unification with Elsevier’s Scopus’ main IDs as a starting point

Main name	Main ID	Country	City	Address
X	1			
X	2			
X/Y	3			
	4		A	
	4		B	
	4	i		
	4	ii		
	4			a
	4			b

### Conceptualizing organizations: formulating rules to unify strings

In unifying the main organization IDs we introduce a threefold rule. Organization id “X” and organization id “Y” pertain to the same unique organization if:

1. if both belong to the same meta-organization (we call this the hierarchical rule) and,
2. if both belong to the same institutional sphere and the same institutional sphere as the meta-organization (we call this the institutional rule) and,
3. if both belong to the same geographical region in which they are not further apart from each other than 50 km (we call this the geographical rule).

Note that this threefold rule only applies to unifying the different main organization IDs. However, in assigning every main name to a particular hierarchy we will split those strings. In the example from Table 3 given above we will split main ID 3 (referring to both X and Y) into main ID 3.1 (X) and main ID 3.2 (Y). Hence, with respect to the main names, the resulting list of strings will only involve a problem of unification (although on the basis of the geographical rule main IDs might still be split!).

### Classifying organizations (II): applying the classification rules

In order to apply the rules thus defined we made use of two additional sources. One source is the organizations’ websites that we found using the text of the longest main name of every unique main ID. Searching for these websites we could first of all assess whether the text of a main name refers to a single organization (e.g., X) or to multiple organizations (e.g., X and Y). As argued earlier, once a single name refers to multiple organizations we split the string (3) into multiple strings (3.1 and 3.2). Second, from each website we assessed whether the organization thus addressed is part of a larger (meta) organization. If so, we noted the website of hierarchical levels. For example, “Harvard Medical School” is part of “Harvard University”; hence we noted both http://hms.harvard.edu/hms/home.asp and http://www.harvard.edu/. Third, from each website we noted the institutional domain of the particular (meta) organization. We looked for the mission statements mentioned on the organizations’ websites. On the basis of these mission statements we assigned every (meta) organization to a particular institutional domain. Similar to Parsons’ (1956a, b) idea of bracketing up society into sub-domains we distinguish among four such institutional domains: industry, care, academia, and political.

Table 4 summarizes the rationale for assigning organizations to a particular institutional domain. Whenever an organization does not mention a mission statement on their website (as e.g., Harvard University!), we assigned them an institutional domain on the basis of their names (hence Harvard University has been assigned to academia). Note that we assigned university hospitals to the institutional domain of care rather than academia. In light of our concern with new modes of knowledge production in which the involvement of non-academic organizations is stressed, we believe that taking university hospitals as performing different activities than universities is legitimate. Finally, we merged those main IDs that belong to both the same meta-organization and the same institutional sphere.

**Table 4:** Assigning organization level names to institutional domains

Institutional domain	Mission	Examples
Industry	Prime objective of generating income or profit for its owners	Pharmaceutical companies; consultants; insurance companies
Care	Prime objective of providing medical care	Hospitals; medical centers (both private and public; including university medical centers)
Academia	Prime objective of producing medical scientific knowledge without profit orientation	Universities; schools; research institutes (both private and public; not-for-profit or non-profit)
Political	Prime objective proposing medical policy and its enforcement	NIH; ministries (public); WHO; NHS; patient representative groups

The other source that we used in applying the rules defined previously (“Conceptualizing organizations: formulating rules to unify strings” of this Appendix), is an online tool to geocode information on the location of organizations (http://www.gpsvisualizer.com/geocoding.html; see also Leydesdorff and Persson 2010). First, from every string we group all three named entities that contain information of the location of the organization level information string. As such, we created a new named entity containing information like “address, city/region, country” and geocoded these new named entities accordingly. For every pair of strings that belong to the same hierarchy and the same institutional domain but have been assigned different geographical coordinates we calculated the kilometer distance separating them. From these distances and using K-means clustering we grouped all strings that are within a range of 50 km from each other and attributed a new coordinate (longitude, latitude) to this organization. We use 50 km as a reasonable range whereas figures on labor commuting areas revolve on this number (see e.g., Karlsson and Olsson 2006). Apart from taking 50 km we also experimented with 30 and 70 km as our geographical boundary of the organization. These alternative geographical boundaries did not alter the results of our main analyses (see Hardeman et al. 2012). In all then, following this threefold procedure we unified all main IDs that occur more than 9 times in our data set and on a global level eventually end up with 1,218 distinct organizations that can be characterized as a coordinate in five-dimensional space.

## Footnotes

1 The classic contribution to this debate is probably Coase (1937) “The nature of the firm”. Theories of the firm have been formulated ever since; ranging from transaction costs (Williamson 1981) to knowledge based theories (Grant 1996) and from a resource based view (Wernerfelt 1984) to a capabilities approach (Teece et al. 1997). Some of these approaches will be discussed in “The logics of the boundary of the organization” of this paper.

2 The examples considered here are drawn from Hjørland and Nissen Pedersen (2005) comparison of objects with different shapes and colors.

3 For example the knowledge base as delineating organizational boundaries (Nonaka 1994; Grant 1996; Teece et al. 1997).

4 Note that our interest presents an example of top-down scientometric research par excellence. That is, going from all the publications on a particular topic the main task at hand revolves singling out all organizations from the complete set of publications thus available. At the same time this means that we did not have to find all publications belonging for example to a single company (including those of its subsidiaries) as is the main problem for bottom-up scientometric research.

5 It could be argued that instead of organizations our study is about the system of organization branches. Although we are appreciative to this point we do not think it alters our argument.

## References

[CR1] Badaracco JJ, Etzioni A, Lawrence PR (1991). The boundaries of the firm. Socio-economics: toward a new synthesis.

[CR2] Bernstein RJ (2010). The pragmatic turn.

[CR3] Bonaccorsi A (2008). Search regimes and the industrial dynamics of science. Minerva.

[CR4] Bourke P, Butler L (1996). Standards issues in a national bibliometric database: the Australian case. Scientometrics.

[CR5] Bourke P, Butler L (1998). Institutions and the map of science: matching university departments and fields of research. Research Policy.

[CR6] Bowker GC, Star SL (1999). Sorting things out: classification and its consequences.

[CR7] Carlile PR (2004). Transferring, translating, and transforming: an integrative framework for managing knowledge across boundaries. Organization Science.

[CR8] Carlsson B, Jacobsson S, Holmén M, Rickne A (2002). Innovation systems: analytical and methodological issues. Research Policy.

[CR9] Coase RH (1937). The nature of the firm. Economica.

[CR10] Collins HM (1985). Changing order: replication and induction in scientific practice.

[CR11] Collins HM (2009). We cannot live by scepticism alone. Nature.

[CR12] De Bruin RE, Moed HF, Egghe L, Rousseau R (1990). The unification of addresses in scientific publications. Informetrics 89/90.

[CR13] Dewey J (1929). The quest for certainty: a study of the relation of knowledge and action.

[CR14] Dicken P, Malmberg A (2001). Firms in territories: a relational perspective. Economic Geography.

[CR15] Elsevier Pharma Development Group (2009). EMTREE: The life Science thesaurus. Elsevier Version 8.0.

[CR16] Etzkowitz H, Leydesdorff L (2000). The dynamics of innovation: from national systems and “mode 2” to a triple helix of university-industry-government relations. Research Policy.

[CR17] Frenken K, Hardeman S, Hoekman J (2009). Spatial scientometrics: towards a cumulative research agenda. Journal of Informetrics.

[CR18] Galvez C, Moya-Anegón F (2006). The unification of institutional addresses applying parametrized finite-state graphs (P-FSG). Scientometrics.

[CR19] Galvez C, Moya-Anegón F (2007). Standardizing formats of corporate source data. Scientometrics.

[CR20] Gibbons M, Limoges C, Nowotny H, Schwartzman S, Scott P, Trow M (1994). The new production of knowledge: the dynamics of science and research in contemporary societies.

[CR21] Grant RM (1996). Toward a knowledge-based theory of the firm. Strategic Management Journal.

[CR22] Hardeman S, Frenken K, Nomaler Ö, Ter Wal A (2012). A proximity approach to the comparative analysis of innovation systems.

[CR23] Hessels L, Van Lente H (2008). Re-thinking new knowledge production: a literature review and a research agenda. Research Policy.

[CR24] Hjørland B (2008). Core classification theory: a reply to Szostak. Journal of Documentation.

[CR25] Hjørland B, Nissen Pedersen K (2005). A substantive theory of classification for information retrieval. Journal of Documentation.

[CR26] Hood WW, Wilson CS (2003). Informetric studies using databases: opportunities and challenges. Scientometrics.

[CR27] Ingwersen O, Christensen FH (1997). Data set isolation for bibliometric online analyses of research publications: fundamental methodological issues. Journal of the American Society for Information Science.

[CR28] Karlsson C, Olsson M (2006). The identification of functional regions: theory, methods, and applications. Annals of Regional Science.

[CR29] Larsen PO (2008). The state of the art in publication counting. Scientometrics.

[CR30] Leydesdorff L, Persson O (2010). Mapping the geography of science: distribution patterns and networks of relations among cities and institutes. Journal of the American Society for Information Science and Technology.

[CR31] Lundvall B, Dosi G, Freeman C, Nelson R, Silverberg G, Soete L (1988). Innovation as an interactive process: from user-producer interaction to the national system of innovation. Technical change and economic theory.

[CR32] Lundvall B (2007). National innovation systems: analytical concept and development tool. Industry and Innovation.

[CR33] Mai J (2004). Classification in context: relativity, reality, and representation. Knowledge Organization.

[CR34] Mäki U, Salanti A, Screpanti E (1997). The one world and the many theories. Pluralism in economics: new perspectives in history and methodology.

[CR35] McGrath WE (1996). The unit of analysis (objects of study) in bibliometrics and scientometrics. Scientometrics.

[CR36] Mizruchi MS, Schwartz M (1992). Intercorporate relations: the structural analysis of business.

[CR37] Moed HF, Egghe L, Rousseau R (1988). The use of on-line databases for bibliometric analysis. Informetrics 87/88.

[CR38] Moed HF (2005). Citation analysis in research evaluation.

[CR39] Moed HF, De Bruin RE, Van Leeuwen TH (1995). New bibliometric tools for the assessment of national research performance: database description, overview of indicators and first applications. Scientometrics.

[CR40] Morgan G (1986). Images of Organization.

[CR41] Nonaka I (1994). A dynamic theory of organizational knowledge creation. Organization Science.

[CR42] Nowotny H, Scott P, Gibbons M (2001). Rethinking science: knowledge and the public in an age of uncertainty.

[CR43] Opthof T, Leydesdorff L (2010). *Caveats* for the journal and field normalizations in the CWTS (“Leiden”) evaluations of research performance. Journal of Informetrics.

[CR44] Parsons T (1956). Suggestions for a sociological approach to the theory of organizations—I. Administrative Science Quarterly.

[CR45] Parsons T (1956). Suggestions for a sociological approach to the theory of organizations—II. Administrative Science Quarterly.

[CR46] Peirce CS, Talisse RB, Aikin SF (1868). Some consequences of four incapacities. The pragmatism reader: from Peirce through the present.

[CR47] Putnam H (2002). The collapse of the fact/value dichotomy.

[CR48] Rip A, Van der Meulen BJR (1996). The post-modern research system. Science and Public Policy.

[CR49] Shapin S (1992). Why the public ought to understand science-in-the-making. Public Understanding of Science.

[CR50] Sher IH, Garfield E, Elias AW (1966). Control and elimination of errors in ISI services. Journal of Chemical Documentation.

[CR51] Shleifer A, Vishny RW (1997). A survey of corporate governance. Journal of Finance.

[CR52] Smith LC (1981). Citation analysis. Library Trends.

[CR53] Spärck Jones K (2005). Some thoughts on classification for retrieval. Journal of Documentation.

[CR54] Steno Diabetes Center (2011). http://www.stenodiabetescenter.com/documents/home_page/document/index.asp. Accessed 20 February 2011.

[CR55] Tattersall R (2009). Diabetes: the biography.

[CR56] Teece DJ, Pisano G, Shuen A (1997). Dynamic capabilities and strategic management. Strategic Management Journal.

[CR57] U.S. National Library of Medicine (2011). Medical subject headings (MeSH). http://www.nlm.nih.gov/mesh/meshhome.html. Accessed 4 February 2011.

[CR58] Van Raan AFJ (2005). Fatal attraction: conceptual and methodological problems in the ranking of universities by bibliometric methods. Scientometrics.

[CR59] Van Raan AFJ (2005). For your citations only? Hot topics in bibliometric analysis. Measurement: Interdisciplinary Research & Perspective.

[CR60] Weingart P (2005). Impact of bibliometrics upon the science system: inadvertent consequences?. Scientometrics.

[CR061] Wernerfelt B (1984). A resource based view of the firm. Strategic Management Journal.

[CR61] Williamson OE (1981). The economics of organization: the transaction cost approach. American Journal of Sociology.

[CR62] World Health Organization (2011). *ICD-10: International statistical classification of diseases and related health problems* (Vol. 2). 10th Revision, Instruction Manual, 2010 edition.

